# Supplementary material for: Cost-effectiveness analysis of implementing screening on preterm pre-eclampsia at first trimester of pregnancy in Germany and Switzerland
Source: PLoS One. 2022 Jun 28;17(6):e0270490. doi: 10.1371/journal.pone.0270490 (PMC9239465; doi:10.1371/journal.pone.0270490)
Supplement: S1 Table — (DOCX) [file pone.0270490.s001.docx]

**Supporting information**

Dataset

**S1A Table. Full input data for Germany**

| **Parameter** | **Base case value** | **Lower limit** | **Upper limit** | **Range used*** | **Distribution used for PSA** | **Standard error** |
| --- | --- | --- | --- | --- | --- | --- |
| Estimated number of pregnancies | 763,732 |  |  |  | Gamma | 76,373 |
| **Parameters routine screening** |  |  |  |  |  |  |
| Probability of PE <37 weeks in routine screening | 0.72% | 0.54% | 0.90% | +/- 25% | Beta | 0.07% |
| Probability of being at high risk of PE in routine screening | 20.0% | 15.00% | 25.00% | +/- 25% | Beta | 2.00% |
| Probability of receiving aspirin prophylaxis when at high risk, routine screening | 80.0% | 60.00% | 100.00% | +/- 25% | Beta | 8.00% |
| **Parameters Screening** |  |  |  |  |  |  |
| Probability being at high risk in PE screening | 11.03% | 8.27% | 13.78% | +/- 25% | Beta | 1.10% |
| Probability of receiving low dose aspirin prophylaxis when at high risk in PE screening | 100% | 75.00% | 125.00% | +/- 25% | Beta | 10.00% |
| Probability of PE <37 weeks when at high risk, PE screening | 1.63% | 1.22% | 2.04% | +/- 25% | Beta | 0.16% |
| Probability of PE <37 when not at risk, PE screening | 0.182% | 0.14% | 0.23% | +/- 25% | Beta | 0.02% |
| **Parameters, HAVING preterm PE <37 weeks** |  |  |  |  |  |  |
| Additional LOS mother for PE (days), with PE | 3.00 | 2.00 | 5.00 | Range | Gamma | 0.30 |
| LOS neonate (days), with PE | 16.00 |  |  |  | Gamma | 1.60 |
| Percentage elective c-section, with PE | 92.80% |  |  |  | Beta | 9.28% |
| Percentage emergency c-section, with PE | 6.40% |  |  |  | Beta | 0.64% |
| Percentage vaginal birth, with PE | 0.80% |  |  |  | Beta | 0.08% |
| Probability of stillbirth, with PE | 0.74% | 0.51% | 0.93% | +/- 25% | Beta | 0.07% |
| Probability of preterm birth, with PE | 75.0% | 51.56% | 93.75% | +/- 25% | Beta | 7.50% |
| Number of quartiles of pregnancy check-up, with PE | 3.00 |  |  |  | Gamma | 0.30 |
| Additional visits to outpatient clinic after delivery, with PE | 1.00 | 0.00 | 3.00 | Range | Gamma | 0.10 |
| Percentage of women attending follow-up at gynaecologist, with PE | 90% | 65.00% | 100.00% | +/- 25% | Beta | 9.00% |
| **Parameters NOT HAVING PE <37 weeks** |  |  |  |  |  |  |
| Average LOS mother (days), no PE | 3.53 | 2.00 | 5.00 | Range | Gamma | 3.43 |
| Percentage c-section, no PE | 29.10% | 20.01% | 36.38% | +/- 25% | Beta | 0.29 |
| Percentage vaginal birth, no PE | 70.90% |  |  |  | Beta | 0.75 |
| Probability of preterm birth, no PE | 8.82% | 6.06% | 11.03% | +/- 25% | Beta | 0.10 |
| Probability stillbirth, no PE | 0.36% | 0.25% | 0.45% | +/- 25% | Beta | 0.00 |
| Number of quartiles of pregnancy check-up, no PE | 3.00 |  |  |  | Gamma | 2.85 |
| Additional visits to outpatient clinic after delivery, no PE | 1.00 | 1.00 | 2.00 | +/- 25% | Gamma | 1.19 |
| Percentage of women attending follow-up at gynaecologist, no PE | 90% | 65.00% | 100.00% | +/- 25% | Beta | 0.98 |
| **Cost parameters** |  |  |  |  |  |  |
| Cost PE screening | 90 € | 60 € | 120 € | Range | Gamma | 87.71 |
| Cost low dose aspirin started at week 14 | 11 € | 8 € | 14 € | +/- 25% | Gamma | 10.79 |
| Cost c-section | 3,444 € | 2,368 € | 4,305 € | +/- 25% | Gamma | 3611.96 |
| Cost vaginal birth | 2,101 € | 1,444 € | 2,626 € | +/- 25% | Gamma | 2073.86 |
| Cost NICU, per day | 1,536 € | 1,056 € | 1,920 € | +/- 25% | Gamma | 1308.20 |
| Cost general ward, per day | 1,171 € | 805 € | 1,464 € | +/- 25% | Gamma | 1260.76 |
| Cost stillbirth | 1,516 € | 1,042 € | 1,895 € | +/- 25% | Gamma | 1164.94 |
| Cost hospital stay healthy newborn | 704 € | 484 € | 880 € | +/- 25% | Gamma | 709.62 |
| Cost hospital stay preterm neonate | 22,257 € | 15,302 € | 27,822 € | +/- 25% | Gamma | 22528.86 |
| Cost additional healthcare of preterm neonate after discharge | 1,675 € | 1,152 € | 2,094 € | +/- 25% | Gamma | 1647.28 |
| Cost check-ups of women per quarter of a year | 130 € | 89 € | 163 € | +/- 25% | Gamma | 127.37 |
| Cost additional healthcare mother after birth when having PE | 130 € | 89 € | 163 € | +/- 25% | Gamma | 136.53 |

PE: preeclampsia, PSA: probabilistic sensitivity analysis

*) When no range is provided, the parameter was not included in the one-way sensitivity analysis.

All sources are listed and references in table 1 in the main manuscript.

**S1A Table. Full input data for Switzerland**

| **Parameter** | **Base case value** | **Lower limit** | **Upper limit** | **Range used*** | **Distribution used for PSA** | **SE** |
| --- | --- | --- | --- | --- | --- | --- |
| Number of births annual | 86,172 |  |  |  | Gamma | 8,617.20 |
| Twin rate | 0.0164 |  |  |  | Beta | 0.00 |
| Estimated number of pregnancies | 84,759 |  |  |  | Gamma | 8,475.88 |
| **Parameters routine screening** |  |  |  |  |  |  |
| Probability of PE <37 weeks in routine screening | 0.72% | 0.50% | 0.90% | +/-25% | Beta | 0.00 |
| Probability of being at high risk of PE in routine screening | 10.00% | 6.88% | 12.50% | +/-25% | Beta | 0.01 |
| Probability of receiving aspirin prophylaxis when at high risk, S routine screening | 50.00% | 34.38% | 62.50% | +/-25% | Beta | 0.05 |
| **Parameters PE screening** |  |  |  |  |  |  |
| Probability being at high risk in PE screening | 11.03% | 7.58% | 13.78% | +/-25% | Beta | 0.01 |
| Probability of receiving low dose aspirin prophylaxis when at high risk in PE screening | 100.00% | 75.00% | 100.00% | +/-25% | Beta | 0.10 |
| Probability of PE <37 weeks when at high risk, PE screening | 1.63% | 1.12% | 2.04% | +/-25% | Beta | 0.00 |
| Probability of PE <37 when not at risk, PE screening | 0.18% | 0.13% | 0.23% | +/-25% | Beta | 0.00 |
| **With preterm PE** |  |  |  |  |  |  |
| Percentage c-section, with PE | 61.88% | 42.54% | 77.35% | +/-25% | Beta | 0.06 |
| Percentage vaginal birth, with PE | 38.12% |  |  |  | Beta | 0.04 |
| Probability of stillbirth, with PE | 0.74% | 0.51% | 0.93% | +/-25% | Beta | 0.00 |
| Probability of preterm birth, with PE | 75.00% | 48.04% | 93.75% | +/-25% | Beta | 0.08 |
| Number of visits to gynaecologist during pregnancy, with PE | 7 | 5 | 9 | +/-25% | Gamma | 0.70 |
| Number of ultrasounds during pregnancy, with PE | 2 | 1 | 3 | Range | Gamma | 0.20 |
| Number of visits to outpatient clinic after delivery, with PE | 1 | 1 | 2 | Range | Gamma | 0.10 |
| **With preterm PE** |  |  |  |  |  |  |
| LOS mother (days) after giving birth, no PE | 5.40 | 4.60 | 6.20 | +/-25% | Gamma | 0.54 |
| Percentage elective c-section, no PE | 17.80% |  |  |  | Beta | 0.02 |
| Percentage emergency c-section, no PE | 16.40% |  |  |  | Beta | 0.02 |
| Percentage vaginal birth, no PE | 65.80% |  |  |  | Beta | 0.07 |
| Probability of preterm birth, no PE | 6.70% | 5.03% | 2.04% | +/-25% | Beta | 0.01 |
| Probability of stillbirth, no PE | 0.36% | 0.27% | 0.45% | +/-25% | Beta | 0.00 |
| Number of visits to gynaecologist during pregnancy, no PE | 7 | 4.8 | 8.8 | Range | Gamma | 0.70 |
| Number of ultrasounds during pregnancy, no PE | 2 | 1.4 | 2.5 | Range | Gamma | 0.20 |
| Visits to outpatient clinic after delivery, no PE | 1 | 0.5 | 2.0 | Range | Gamma | 0.10 |
| **Costs** |  |  |  |  |  |  |
| Cost PE screening | 150 CHF | 110 CHF | 190 CHF | Range | Gamma | 15.00 |
| Cost PLGF | 80 CHF |  |  |  | Gamma | 8.00 |
| Cost ultrasound | 70 CHF |  |  |  | Gamma | 7.00 |
| Cost low dose aspirin started at week 14 | 23 CHF | 17 CHF | 29 CHF |  | Gamma | 2.29 |
| Cost c-section | 9,269 CHF | 6,952 CHF | 11,586 CHF |  | Gamma | 926.89 |
| Cost vaginal birth | 6,158 CHF | 4,619 CHF | 7,698 CHF |  | Gamma | 615.83 |
| Cost additional days at hospital (mother) when having PE | 3,986 CHF | 2,990 CHF | 4,983 CHF |  | Gamma | 398.64 |
| Cost admission healthy new-born | 2,133 CHF | 1,600 CHF | 2,666 CHF |  | Gamma | 213.30 |
| Cost stillbirth | 2,417 CHF | 1,813 CHF | 3,022 CHF |  | Gamma | 241.74 |
| Cost hospital stay preterm neonate | 67,837 CHF | 50,878 CHF | 84,797 CHF |  | Gamma | 6,783.73 |
| Cost visit to gynaecologist during pregnancy | 36 CHF | 27 CHF | 45 CHF |  | Gamma | 3.57 |
| Cost additional healthcare of preterm neonate after discharge | 1,855 CHF | 1,391 CHF | 2,319 CHF |  | Gamma | 185.50 |
| Cost ultrasound during pregnancy | 142 CHF | 106 CHF | 177 CHF |  | Gamma | 14.19 |
| Cost visit to outpatient clinic after delivery | 49 CHF | 37 CHF | 61 CHF |  | Gamma | 4.91 |

PE: preeclampsia, PSA: probabilistic sensitivity analysis

*) When no range is provided, the parameter was not included in the one-way sensitivity analysis.

All sources are listed and references in table 1 in the main manuscript.

Values used to build graphs

**S3a Table. Input data for tornado diagram of one-way sensitivity analysis for Switzerland.**

| **Parameter** | **Higher value** | **Lower value** | **Absolute difference** |
| --- | --- | --- | --- |
| Cost PE screening | 44.36 | -15.64 | 60.00 |
| Probability of PE <37 weeks in routine screening | -21.34 | 23.29 | 44.62 |
| Probability of preterm birth, with PE | -2.12 | 23.64 | 25.76 |
| LOS neonate (days), with PE | -1.03 | 23.02 | 24.05 |
| Cost NICU, per day | 0.89 | 21.94 | 21.05 |
| Probability of PE <37 weeks when at high risk, PE screening | 23.27 | 9.35 | 13.92 |
| Additional LOS mother for PE (days), with PE | 5.50 | 18.80 | 13.30 |
| Probability being at high risk in PE screening | 22.59 | 9.74 | 12.85 |
| Probability of PE <37 when not at risk, PE screening | 22.39 | 9.85 | 12.54 |
| Cost general ward, per day | 11.04 | 16.23 | 5.19 |

PE: preeclampsia, LOS: length of stay, NICU: neonatal intensive care unit

**S4a Table. Input data for tornado diagram of one-way sensitivity analysis for Switzerland.**

| **Parameter** | **Higher value** | **Lower value** | **Absolute difference** |
| --- | --- | --- | --- |
| Probability of PE <37 weeks in routine screening | -133.46 | 9.98 | 143.44 |
| Probability of preterm birth, with PE | -89.61 | 27.28 | 116.89 |
| Cost PE screening | -1.66 | -81.66 | 80.00 |
| Cost hospital stay preterm neonate | -85.50 | -30.70 | 54.80 |
| Probability of PE <37 weeks when at high risk, PE screening | -18.75 | -54.54 | 35.79 |
| Probability being at high risk in PE screening | -20.68 | -53.46 | 32.78 |
| Probability of PE <37 when not at risk, PE screening | -21.03 | -53.27 | 32.24 |
| Percentage vaginal birth, no PE | -37.83 | -42.62 | 4.79 |
| Cost additional days at hospital (mother) when having PE | -45.43 | -40.72 | 4.72 |
| Cost c-section | -44.09 | -41.05 | 3.03 |

PE: preeclampsia, LOS: length of stay, NICU: neonatal intensive care unit

**S5a Table. Input data for cost-effectiveness acceptability curve for willingness-to-pay for a preterm PE case avoided for Germany**

| **Ceiling ratio [EUR]** | **Probability PE screening most cost-effective** | **Probability routine screening most cost-effective** |
| --- | --- | --- |
| 0 | 0.232 | 0.768 |
| 50 | 0.234 | 0.766 |
| 100 | 0.238 | 0.762 |
| 200 | 0.245 | 0.755 |
| 300 | 0.249 | 0.751 |
| 500 | 0.261 | 0.739 |
| 700 | 0.273 | 0.727 |
| 900 | 0.285 | 0.715 |
| 1,000 | 0.289 | 0.711 |
| 1,200 | 0.300 | 0.700 |
| 1,400 | 0.316 | 0.684 |
| 1,600 | 0.330 | 0.670 |
| 1,800 | 0.342 | 0.658 |
| 2,000 | 0.354 | 0.646 |
| 2,500 | 0.382 | 0.618 |
| 3,000 | 0.418 | 0.582 |
| 3,500 | 0.453 | 0.547 |
| 4,000 | 0.486 | 0.514 |
| 4,500 | 0.521 | 0.479 |
| 5,000 | 0.551 | 0.449 |
| 5,500 | 0.579 | 0.421 |
| 6,000 | 0.609 | 0.391 |
| 7,000 | 0.667 | 0.333 |
| 8,000 | 0.722 | 0.278 |
| 9,000 | 0.770 | 0.230 |
| 10,000 | 0.809 | 0.191 |
| 15,000 | 0.927 | 0.073 |
| 20,000 | 0.970 | 0.030 |
| 25,000 | 0.989 | 0.011 |
| 30,000 | 0.996 | 0.004 |

PE: preeclampsia

**S6a Table. Input data for cost-effectiveness acceptability curve for willingness-to-pay for a preterm PE case avoided for Germany**

| **Ceiling ratio [CHF]** | **Probability PE screening most cost-effective** | **Probability routine screening most cost-effective** |
| --- | --- | --- |
| 0 | 0.787 | 0.213 |
| 50 | 0.788 | 0.212 |
| 100 | 0.789 | 0.211 |
| 200 | 0.790 | 0.210 |
| 300 | 0.793 | 0.207 |
| 500 | 0.797 | 0.203 |
| 700 | 0.800 | 0.200 |
| 900 | 0.804 | 0.196 |
| 1,000 | 0.807 | 0.193 |
| 1,200 | 0.810 | 0.190 |
| 1,400 | 0.813 | 0.187 |
| 1,600 | 0.816 | 0.184 |
| 1,800 | 0.820 | 0.180 |
| 2,000 | 0.825 | 0.175 |
| 2,500 | 0.832 | 0.168 |
| 3,000 | 0.839 | 0.161 |
| 3,500 | 0.847 | 0.153 |
| 4,000 | 0.855 | 0.145 |
| 4,500 | 0.863 | 0.137 |
| 5,000 | 0.869 | 0.131 |
| 5,500 | 0.878 | 0.122 |
| 6,000 | 0.884 | 0.116 |
| 7,000 | 0.895 | 0.105 |
| 8,000 | 0.906 | 0.094 |
| 9,000 | 0.918 | 0.082 |
| 10,000 | 0.928 | 0.072 |
| 15,000 | 0.963 | 0.037 |
| 20,000 | 0.980 | 0.020 |
| 25,000 | 0.989 | 0.011 |
| 30,000 | 0.993 | 0.007 |

PE: preeclampsia
