# Supplementary material for: Cost-effectiveness analysis of implementing screening on preterm pre-eclampsia at first trimester of pregnancy in Germany and Switzerland
Source: PLoS One. 2022 Jun 28;17(6):e0270490. doi: 10.1371/journal.pone.0270490 (PMC9239465; doi:10.1371/journal.pone.0270490)
Supplement: Attachment — Submitted filename: Response to reviewers comments_Mewes et al_21052022.docx [file pone.0270490.s003.docx]

**Response to reviewer´s comments**

PONE-D-21-23946R2

Cost-effectiveness analysis of implementing screening on preterm pre-eclampsia at first trimester of pregnancy in Germany and Switzerland

PLOS ONE

**Review Comment to the Author:**

**Reviewer #1: The authors have addressed my questions and comments very rigorously. The main text, figures and supporting information are clear and easy to follow now.
One last minor point: pls clarify the [+] symbol in Figure 1 (decision tree). As mentioned in a previous comment, I assume that the symbol indicates that the branches following the standard check- up need to be added here. I am not entirely sure however, because of the different terminology: regular ckeck up vs. standard check up. There is still no legend included the figure.**

Thank you for confirming that we have addressed your questions and comments. Regarding the last minor point and the legend for Figure 1; these were addressed in the revised manuscript, page 5, lines 114-115:

[+]: The branches from above are repeated.

PE: preeclampsia, WGA: weeks of gestation.
